# Authors' Reply to “Questioning the Rhythm: A Closer Look at Heart Rate Trends”

**DOI:** 10.1002/clc.70158

**Published:** 2025-06-02

**Authors:** Eitaro Kodani, Takeshi Yamashita, Hiroshi Inoue, Hirotsugu Atarashi, Ken Okumura, Hideki Origasa, J‐RHYTHM Registry Investigators

**Affiliations:** ^1^ Department of Cardiovascular Medicine Nippon Medical School Tama Nagayama Hospital Tokyo Japan; ^2^ The Cardiovascular Institute Tokyo Japan; ^3^ Saiseikai Toyama Hospital Toyama Japan; ^4^ Nippon Medical School Tokyo Japan; ^5^ Saiseikai Kumamoto Hospital Kumamoto Japan; ^6^ The Institute of Statistical Mathematics Tokyo Japan

**Keywords:** adverse event, all‐cause death, atrial fibrillation, changing pattern, heart rate

## 1 The Authors' Reply

We would like to thank Dr. Hassan for your interest in our recently published study “Association between changes in heart rate and adverse events in patients with non‐valvular atrial fibrillation: A post hoc analysis of the J‐RHYTHM Registry.” [1] There were several limitations in our study as already mentioned in the article [1]. Our replies to the comments are as follows.

First, we agree that heart rate (HR) fluctuation may have influenced the incidence of adverse events. However, the present study was based on the results of our previous analysis that HR at the time closest to an event or at the last visit during the follow‐up period (HR‐end) was more strongly associated with the incidence of adverse events than the baseline HR in patients with nonvalvular atrial fibrillation (AF) [2]. Specifically, the highest quartile of HR‐end (≥ 80 bpm) was independently associated with the incidence of major hemorrhage, all‐cause death, and cardiovascular death compared with the second quartile (64–71 bpm), even after adjusting for known confounding factors and HR‐controlling drug use [2]. Therefore, we adopted the changes in HR from the baseline to the end instead of HR fluctuation during the whole follow‐up period in the present analysis [1]. We had HR data at each time when patients visited their outpatient clinic. We could have analyzed visit‐to‐visit changes in HR for each patient; however, for simplicity, we analyzed changes in HR from the baseline to the end of follow‐up [1].

In addition, when patients were divided into four baseline HR groups (< 60, 60–79, 80–109, and ≥ 110 bpm) using clinically relevant cutoff HR values based on the Rate Control Efficacy in Permanent Atrial Fibrillation II (RACE II) trial [3], no significant trend in the rate of any adverse event was observed across these baseline HR groups in our cohort [2]. Therefore, we decided to use the HR quartiles in the present study [1].

Second, as you pointed out, changes in drugs including oral anticoagulants and HR‐controlling drugs, their dosages, and adherence were not considered during the follow‐up period in this study. We recognize this is a limitation of this study. However, hazard ratios were adjusted for the use of HR‐controlling drugs including β‐blocker, K channel blocker, Ca channel blocker, and digitalis in a multivariable Cox regression model in this study [1].

Third, since the J‐RHYTHM Registry was an observational study, the causal relationship between changes in HR and adverse events, as well as the underlying mechanisms, could not be determined from this study. That is known as a general limitation of the observational study. Indeed, we indicated only the association between changes in HR and adverse events and did not mention causality throughout the article. Of course, there is a possibility that the changes in HR are not only the causes of adverse events but also the results of worsening clinical status. Therefore, we proposed in our article [1] that careful management is necessary to identify undiagnosed underlying diseases or hidden unfavorable conditions and prevent subsequent adverse events when patients have excessive increases in HR or consistently high HR.

Fourth, for patients with a higher baseline HR, modest decreases in HR during the follow‐up period were significantly associated with lower mortality in the fully adjusted model in this study. In general, statistical underpower often results in type II error. Therefore, abovementioned our result seems not pertinent to type I error. In addition, our result does not conflict with previous reports that the excessive low HR is independently associated with all‐cause mortality in patients with AF [4, 5].

Finally, we agree that further studies using continuous HR monitoring and longitudinal modeling are necessary to confirm the influence of HR fluctuation on adverse events in patients with AF since the present study is hypothesis‐generating in nature. Most contents of this reply have already been stated in the Limitations of the article [1].

## Conflicts of Interest

E.K. received remuneration from Daiichi‐Sankyo; T.Y. received remuneration from Bayer Healthcare, Bristol‐Myers Squibb, Daiichi‐Sankyo, Nippon Boehringer Ingelheim, Novartis, and Otsuka Pharmaceutical; H.I. Inoue received remuneration from Daiichi‐Sankyo; H.A. received remuneration from Daiichi‐Sankyo; K.O. received remuneration from Boehringer Ingelheim, Bristol‐Myers Squibb, Daiichi‐Sankyo, Johnson & Johnson, and Medtronic; and H.O. received remuneration from Johnson & Johnson.

## Data Availability

The deidentified participant data will be shared on a request basis. Please directly contact the corresponding author to request data sharing.
